# Field and farm-level data on agricultural land use for the European Union

**DOI:** 10.1038/s41597-025-05210-6

**Published:** 2025-06-20

**Authors:** Clemens Jänicke, Kristoffer Ansbak Petersen, Phillip Schmidts, Daniel Müller, Martin Rudbeck Jepsen

**Affiliations:** 1 Leibniz Institute of Agricultural Development in Transition Economies (IAMO), Halle (Saale), Germany; 2 Geography Department, Humboldt-Universität zu Berlin, Berlin, Germany; 3 Integrative Research Institute on Transformations of Human-Environment Systems (IRI THESys), Humboldt-Universität zu Berlin, Berlin, Germany; 4 Section for Geography, Department of Geosciences and Natural Resource Management, University of Copenhagen, Copenhagen, Denmark

**Keywords:** Agriculture, Geography, Databases

## Abstract

We present field-level land-use data from the Integrated Administration and Control System (IACS), featuring extended thematic details on farm-level indicators and organic cultivation. The data cover 19 of 27 EU member states, with annual time series that extend up to 17 years in some member states. The IACS data are invaluable for analysing land-use changes and farm-level characteristics with fine spatial, thematic, and temporal details. We provide an overview of the data sources, outline the pre-processing and harmonisation workflow and provide maps that exemplify the coverage and potential analyses. The data inventory constitutes a living document to which we will add additional data should these become available.

## Background & Summary

Agricultural land use occupies more than one-third of the terrestrial surface, yet a consistent pan-European overview of detailed field-level spatial information on agricultural land use is unavailable. While field-level land-use data exist, they are scattered across national authorities in the EU, often the national paying agencies, and are only accessible in a limited number of member states with varying levels of detail. Remote sensing offers an option for wall-to-wall mapping but measures land cover instead of land use, provides limited information on farm and field sizes, and fails to monitor crucial farming system indicators, such as farm size or cultivation intensity^1^. Inferences can be made from land cover to land use, but some land-cover classes are ambiguous regarding land use, such as grasslands that can be used for grazing, mowed for fodder, or remain uncultivated. Assessing the many subtle differences in land use, such as agricultural intensity within the cropland class, livestock numbers, and whether farming follows conventional or organic cultivation principles, remains challenging with satellite imagery^2^.

In the EU, data from the Integrated Administration and Control System (IACS) offer detailed spatial and thematic information on agricultural land use at the field and farm level^3^. Until now, the data have been used for various analyses at local^4–6^, regional^7–12^, and national scales^13–15^. The increasing number of case studies that examine fine-scale land-use patterns and changes attest to the value of IACS for land-system science.

**Fig. 1:**
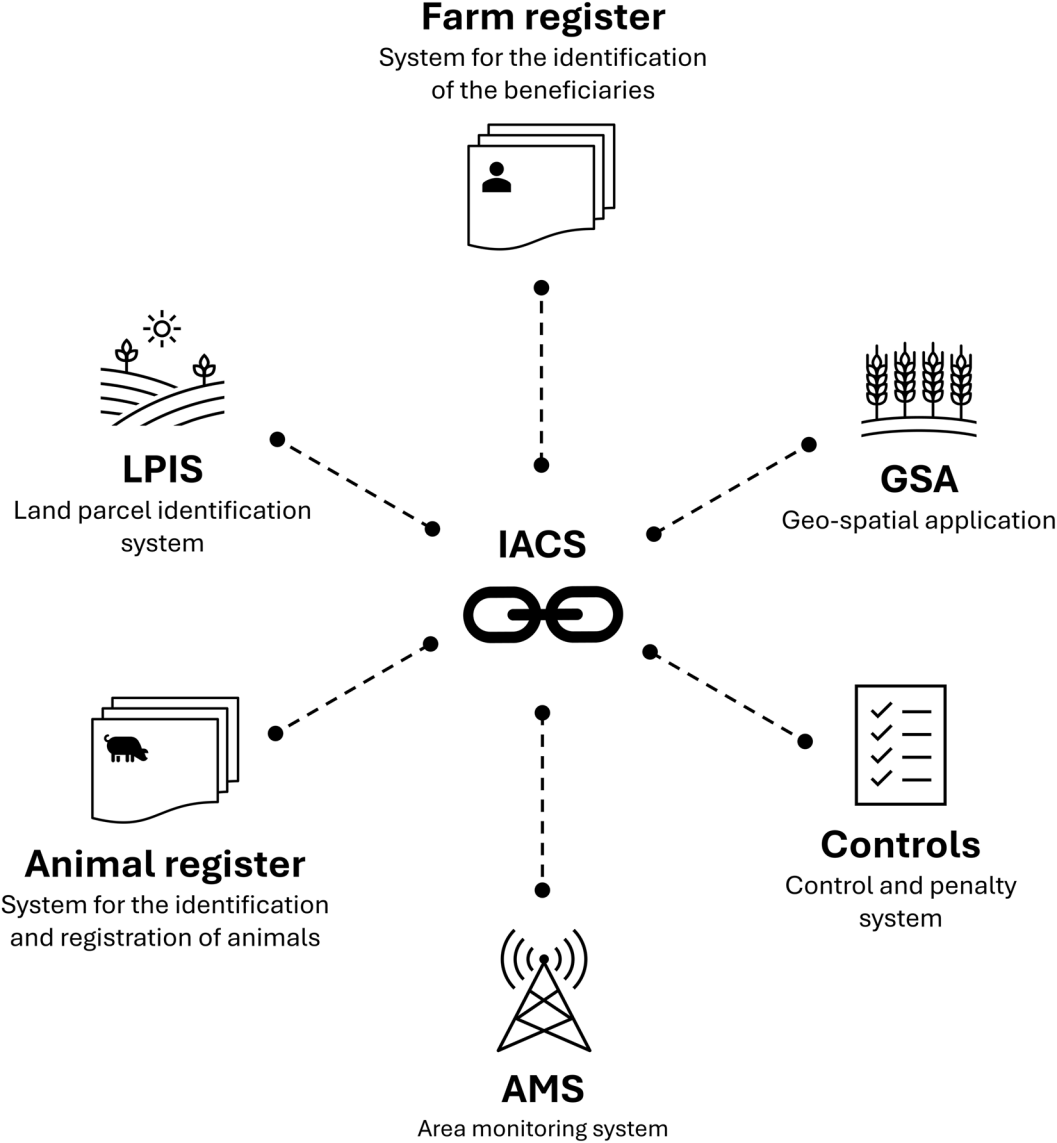
Overview of the digital components, systems, and databases comprising IACS.

The IACS encompasses a variety of interconnected digital elements and databases (Fig. 1). It includes two key geospatial subsystems: the Land Parcel Identification System (LPIS) and the Geospatial Application (GSA). The LPIS identifies agricultural land eligible for payments under the EU’s Common Agricultural Policy (CAP) within designated areas. In IACS terminology, these areas are referred to as reference parcels. Each reference parcel may consist of multiple agricultural parcels, which are the main spatial units represented in the GSA geodata. The agricultural parcels (hereafter referred to as ‘parcels’) are delineations of areas declared by the farmers themselves as part of their subsidy application. The GSA data provide unique land-use information for each parcel for which applicants have applied for subsidies through the CAP in a given year. For some member states, also non-subsidised parcels are registered in the GSA geodata. The Area Monitoring System (AMS) observes and assesses agricultural activities. It aims to reduce administrative burdens in the verification process of the applications and ensures application accuracy by allowing modifications after on-the-spot checks or virtual controls. IACS also includes systems for identifying beneficiaries and registering payment entitlements, a control and penalty system to ensure legal and regular payments, and a system for identifying and registering animals in member states with animal-based interventions (https://agriculture.ec.europa.eu/common-agricultural-policy/financing-cap/assurance-and-audit/managing-payments_en).

The IACS data are administered and managed by federal and state-level authorities in all EU member states. Thus far, it has been challenging to access the data as it sits with the federal or state-level payment agencies or ministries, which are often reluctant to release IACS data for open use. However, data policies are changing, not least thanks to the increasing push by the European Commission to make geospatial data available for scientific purposes (Regulation (EU) 2021/2116, 2021).

In the Horizon Europe project Europe-LAND (https://europe-land.eu), we searched for parcel-level crop information from GSA geodatasets for all 27 EU member states. We obtained, harmonised, and published GSA data for 19 EU member states in our data inventory. In addition, we have GSA data for six more member states (Italy, Greece, Poland, Hungary, Romania, and Cyprus), which we currently cannot share due to General Data Protection Regulations (GDPR) and data-sharing restrictions. Member states for which only LPIS data were available (Luxembourg and Malta) were excluded from the inventory (see Usage Notes for details on data not included in the inventory).

**Fig. 2:**
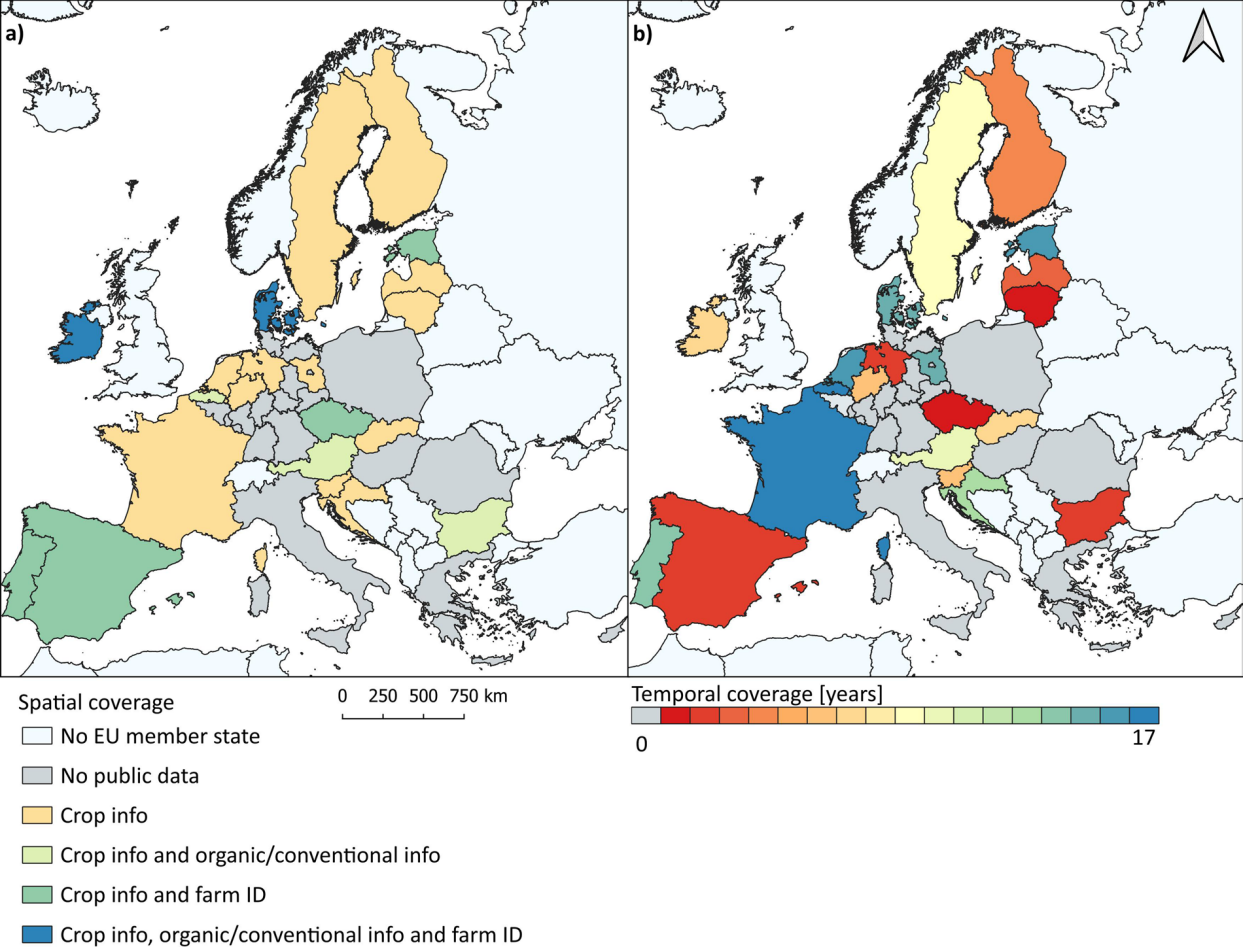
Overview of (**a**) the spatial and thematic coverage regarding crop codes, crop labels, and farm identifiers, and (**b**) the temporal coverage in years of the data inventory. Due to variations in data accessibility within member states, the data for Germany are presented at the state level and for Belgium at the regional level. For all other member states, the data are depicted at the national level.

Our contribution builds on and expands an earlier harmonisation approach focused on parcel-level land-use data by the EuroCrops project^16^. EuroCrops developed a hierarchical crop and agricultural taxonomy (HCAT) to harmonise all crop classes following the EAGLE principles^17^. The HCAT categorises each crop into a ten-digit code and a related readable name. It consists of six nested levels, with higher levels containing broader information on land use and the lowest levels containing crop types. We are in contact with the EuroCrops project team and will apply upcoming revisions of the HCAT to our inventory as the source code becomes available. Our approach substantially expands the EuroCrops efforts in spatial and temporal extent and complements these with thematic details beyond the crop information. We collated farm-level indicators, such as farm identifiers, which are valuable for identifying parcels managed under one farm, allowing, for example, to calculate farm size. Moreover, in cases where the datasets included information on the organic status of a parcel or farm, we include this in the inventory. Since the inventory only presents GSA data, information on the organic status of parcels or farms from other databases are not included. Farm identifiers and organic information data was, unfortunately, only available for a subset of member states (Fig. 2).

We refer readers to the Zenodo hyperlink^18^ for the most recent coverage of the data inventory. We will update the link as additional data becomes available and as new versions of the HCAT are released.

## Methods

### Data collection

Collecting IACS data is tedious due to the varying levels of data accessibility among EU member states. While all EU member states are required to make key elements of the IACS data publicly available for purposes of the Infrastructure for Spatial Information in Europe (INSPIRE) network to create a standardised spatial data infrastructure for the monitoring of EU policies (Regulation (EU) 2021/2116, 2021), these requirements are not always met. For this project, we began the data collection process in July 2023 and continue to add new data to the inventory as these become available.

All GSA geodatasets included in the inventory were retrieved directly from public sources, such as national or regional websites and geoportals typically managed by national paying agencies or ministries. The majority of the collected data depict crop-specific information at the parcel level; however, in some member states, GSA parcel-level information is aggregated at the reference-parcel level. In such cases, multiple crops may be declared per reference parcel. Moreover, for some member states, the data include farm-level indicators and organic farming data. While we tried to acquire the complete data for as many years as possible, this was not feasible for all member states due to various constraints, such as data unavailability, limited accessibility, or lack of responses to our inquiries. The data sources for each member state are listed in Table 1, with further details about the datasets available in the documentation of the data inventory, which we published on Zenodo^18^ along with the data.

**Table 1:** Summary of data sources for each member state.

Member state	Data source
Austria	https://www.opendataportal.at/suche/?typeFilter%5B%5D=&searchterm=INVEKOS&searchin=data
	https://data.europa.eu/data/catalogues/open-data-portal-austria?locale=en&query=invekos&page=1&limit=10
Belgium – Flanders	https://landbouwcijfers.vlaanderen.be/open-geodata-landbouwgebruikspercelen
Bulgaria	https://seu.dfz.bg/drupal/?q=opendata
Croatia	https://www.apprrr.hr/prostorni-podaci-servisi/
Czechia	https://mze.gov.cz/public/portal/mze/farmar/LPIS/export-lpis-rocni-shp
Denmark	https://landbrugsgeodata.fvm.dk/
Estonia	https://data.europa.eu/data/datasets/2fa494d3-3f1d-4f76-9866-bedd005cada6?locale=en
	Download via WFS: https://data.europa.eu/data/datasets/2fa494d3-3f1d-4f76-9866-bedd005cada6?locale=en
Finland	https://www.ruokavirasto.fi/en/about-us/published-datasets/spatial-data-sets/
France	https://geoservices.ign.fr/rpg
Germany–Brandenburg	https://geobroker.geobasis-bb.de/gbss.php?MODE=GetProductInformation&PRODUCTID=996f8fd1-c662-4975-b680-3b611fcb5d1f
Germany–Lower Saxony	https://sla.niedersachsen.de/landentwicklung/LEA/
Germany–North Rhine-Westphalia	https://www.opengeodata.nrw.de/produkte/umwelt_klima/bodennutzung/landwirtschaft/
Ireland	https://opendata.agriculture.gov.ie/organization/bps-and-rural-development-schemes
Latvia	https://www.lad.gov.lv/lv/lauku-registra-dati
Lithuania	http://www.geoportal.lt/geoportal/web/en/national-paying-agency_download#savedSearchId={097F1ABC-AFCB-4015-AFB2-D4C5A973F3D9}&collapsed=true (an account needs to be created, and the data officially ordered)
Netherlands	https://service.pdok.nl/rvo/brpgewaspercelen/atom/v1_0/basisregistratie_gewaspercelen_brp.xml
Portugal	https://www.ifap.pt/isip/ows/ (direct download used for 2011-2019 data)
	WFS used for 2020-2024 data: https://www.ifap.pt/isip/ows/isip.data/wfs
Slovakia	https://data.slovensko.sk/datasety/cc261225-7153-44a3-8ebf-05af207515c9
Slovenia	https://rkg.gov.si/vstop/
Spain	https://www.fega.gob.es/atom/
Sweden	https://jordbruksverket.se/e-tjanster-databaser-och-appar/e-tjanster-och-databaser-stod/kartor-och-gis#h-Laddanerkartskikt

For instances where data can be downloaded through a Web Feature Service (WFS), both the WFS link and the corresponding website linking to the WFS are provided. All links were accessible at the time of publication.

The compiled data inventory includes 19 EU member states with varying levels of spatial and temporal coverage, as well as differences in thematic detail (Fig. 2). In most member states, the data are administered at the national level and cover the entire member state; however, in a few cases, the subsidy applications are managed at sub-national administrative levels, and thus, the data are managed and stored at the level of federal states (Germany), regions (Belgium), or provinces (Spain). For Spain, we provide the data for all provinces, while in Belgium, only data for Flanders is available for sharing. In Germany, we currently present data for three federal states (Brandenburg, Lower Saxony and North Rhine-Westphalia); for the other federal states, we either cannot share data or requests are still pending at the time of writing. One German federal state, Schleswig-Holstein, refused to share the data due to data privacy issues. Furthermore, the three city-states, Berlin, Bremen, and Hamburg, do not collect the data but are included in the datasets of neighbouring federal states. Additionally, in France and Portugal, the spatial availability of the data varies across the years, with more recent data being provided at the national level, while older data were available at the regional level for Portugal and the provincial level for France. For France, we have complete national coverage for all years presented. In contrast, a full national coverage for Portugal is only available from 2020 onwards.

For most member states, only crop-specific information is available for sharing. However, six of the 19 member states (Czechia, Denmark, Estonia, Ireland, Portugal and Spain) provide a farm identifier, and five of them (Austria, Flanders in Belgium, Denmark, Ireland and Bulgaria) disclose information on organic management (Fig. 2a). Temporally, the data coverage varies between one year (2024 for Lithuania) and 17 years (2007–2023 for France and 2008–2024 for Flanders in Belgium). For seven member states, federal states, or regions, we obtained time series of up to five years; for six, up to ten years; for four, up to 15 years; and for another four, up to 17 years (Fig. 2b).

**Fig. 3:**
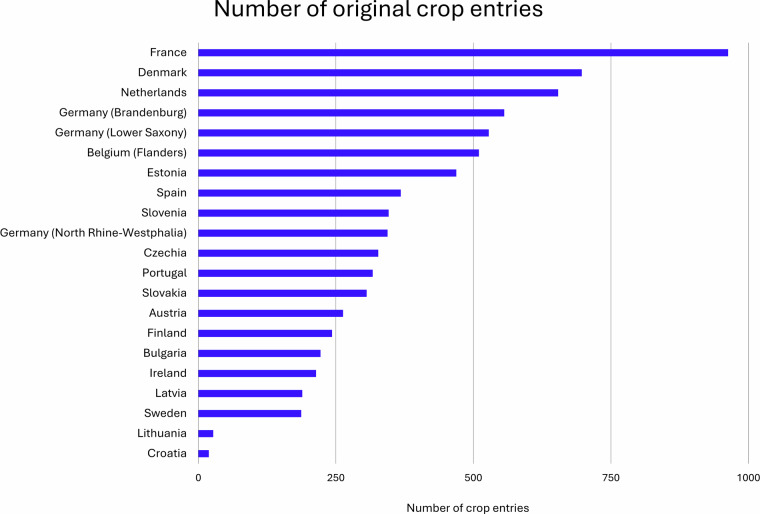
The number of different crop entries of the member states, federal states, and regions within our data inventory.

The original national crop classifications vary from 19 classes in Croatia to 963 classes in France (Fig. 3). The crop classification usually consists of a crop code and an assigned crop name. However, the meaning of crop codes can change between years, crop codes can be added or removed, and the crop names can be written differently in different years. We only reported the unique combinations of crop codes and crop names.

### Pre-processing and harmonisation

To harmonise the datasets across all member states, we developed a uniform data structure of the spatial information and harmonised the information on crops and organic cultivation. The harmonisation was done separately for each member state, federal state, and region. Our harmonisation workflow included the following steps:

First, to explore the data and understand the meaning of data table columns, we listed all column names from the original vector data for each year, along with examples of the values stored in each column. This provided us with an understanding of the data structure and content. Additionally, we manually explored the data in QGIS.

To automate the subsequent steps, we created column-name lookup tables for each member state. This was necessary to automate the assignment of all the original columns to the harmonised column names (Table 2). The table included a separate column with the translation for each year, as column labels varied between years.

**Table 2:** Structure of the harmonised spatial data.

Column name	Column description	Mandatory for harmonisation
field_id	Unique identifier for each parcel per member state, state, or region	Mandatory
farm_id	Unique identifier for each farm per member state, state, or region	Optional
crop_code	Original member state-specific crop code	Mandatory
crop_name	Original member state-specific crop name	Mandatory
EC_trans_n	Original crop name translated into English	Mandatory
EC_hcat_n	Machine-readable name of the crop from HCAT	Mandatory
EC_hcat_c	The ten-digit HCAT code of the hierarchy of the crop	Mandatory
organic	Whether a parcel was cultivated conventional (0), organic (1), or is in the conversion process to organic cultivation (2)	Optional
field_size	Size of parcel/reference parcel in hectares	Mandatory
crop_area	Area in hectares of the main crop reported in the crop column. The crop_area column only occurs if multiple crops are reported per reference parcel.	Optional

Depending on the member state and its data availability, not all columns are present in the harmonised version of the data. The ‘Mandatory for harmonisation’ columns indicate which columns are included in all data and which columns are optional and occur only in selected data. HCAT = Hierarchical Crop and Agricultural Taxonomy.

To harmonise the crops, we listed all available ‘crop code - crop name’ combinations in the vector data. In some cases, the meaning of the crop codes could change between years, or their descriptions could change. Working with the ‘crop code – crop name’ combinations ensured avoiding inconsistencies and misclassifications between the years. We then translated all crop names into English. In cases when a translation was not possible, we conducted a web search using the original crop entry.

For the harmonisation of the crop codes and crop names, we build on the proposed crop harmonisation of the EuroCrops project^16^. Therefore, when available, we used the crop-mapping tables from the EuroCrops project to categorise the ‘crop code – crop name’ combinations (https://github.com/maja601/EuroCrops/tree/main/csvs/country_mappings). In most cases, not all entries could be categorised automatically as the data include crops from multiple years, in contrast to EuroCrops. Hence, we manually expanded the existing EuroCrops tables and categorised all crops that had not been previously categorised. In cases where no crop-mapping table was available from EuroCrops, we manually categorised the crops. To reduce the workload, we used a string-matching algorithm on the English crop names, applying the Jaro-Winkler distance metric to associate unclassified entries with classified entries from other member states. The Jaro-Winkler metric measures string similarity based on the number of matching characters and transpositions (i.e., swapped letters). It is particularly useful for record matching and is therefore well-suited for matching crop type names^19,20^. All matching was performed on the English crop names. Manual verification of the matched entries and classification of any potentially remaining unclassified entries were still necessary; however, the workload was substantially reduced. Once the crop-mapping tables were finalised, we used them to harmonise the crop codes and names into the EuroCrops HCAT.

As described above, the EuroCrops HCAT consists of six hierarchical levels with increasing thematic details, but the original crop classifications of the member states provide different levels of detail. Sometimes, the crop codes of a member state fall into higher levels of the HCAT, while in other cases, they fall into lower levels. We extended the HCAT by including crop-mapping tables of nearly all EU member states (excluding Luxembourg and Malta) and by including historical data, in some cases going back to 2007, into our inventory. To facilitate interoperability with the EuroCrops database, we used the same column names as in the HCAT.

Across member states, there were differences in how the crop-specific information was stored in the data. In some member states, applicants can only declare a single crop at the parcel level, while in others, spatial data are only available at the reference-parcel level. Here, multiple crops can be declared within a reference parcel. When multiple crops were reported per reference parcel, our harmonised geospatial data only contain the crop information of one crop within the reference parcel. In such cases, the ‘field size’ refers to the entire reference-parcel area, while the ‘crop area’ refers to the area of the dominant crop reported in the crop columns. For example, if two crops are reported, with one crop covering 70% of the reference parcel and the other crop covering 30%, then the crop covering 70% is considered the dominant crop, and its area would be reported in the ‘crop area’ column. The additional crop data were saved in a complementary table linked to the reference parcel via the field ID.

Furthermore, we used the column-name translation tables to harmonise the column names. In most cases, the data included a unique national field ID. However, in cases where this ID was missing, we generated a running ID to ensure that all parcels have a unique identifier per member state. Where information was available, we extended the datasets by adding columns for farm identifiers or information on whether a parcel was managed organically. To harmonise the information on organic farming, we classified the parcels into three categories: ‘conventional’, ‘organic’, and ‘in conversion’ using the codes 0, 1, and 2, respectively. In some member states, this information was provided at the parcel level; in others, it was available only at the farm level. For the latter, we assigned the farm-level information to the respective parcels. Future versions of the harmonised inventory will include information on agri-environmental measures, eco-schemes, and animal numbers per farm. All geospatial data were transformed into a projected coordinate system (ETRS89-extended / LAEA Europe – EPSG:3035), and the files were saved as geoparquets.

## Data Records

The data inventory is published on Zenodo^18^, with version 1.2 representing the peer-reviewed version associated with this article. We stored the data as geoparquets, a relatively new geospatial data format (https://geoparquet.org/)^21^. Geoparquets are a spatial data format that extends the Parquet format. Parquet data are in column-storage format with improved storage efficiency through column-oriented compression and encoding and faster processing capabilities. QGIS and various libraries from different programming languages can handle geoparquets (e.g., geoarrow and sfarrow in R, GDAL/OGR in C++, GeoParquet.jl in Julia or Fiona in Python). To comply with the file number limit of Zenodo, we bundled files from multiple years into one zip file per entity, ensuring that the file size of each bundle remained below five GB. Each zip file name indicates the member state, federal state, or region, and the years covered. The meaning of the abbreviations of the member states, federal states, and regions can be found in ‘country_region_codes.xlsx’ in ‘_Documentation.zip’ on Zenodo. Member state codes follow the Interinstitutional Style Guide of the EU (https://style-guide.europa.eu/en/content/-/isg/topic?identifier=annex-a6-country-and-territory-codes). For regions below member state level, i.e. provinces, regions or German *Länder* we assigned a self-defined three-letter code. We also bundled the regional data from Spain, as the data were separated into 50 regions. The crop-mapping tables that are inspired by the EuroCrops project and the tables with column-name translation are available on GitHub (https://github.com/clejae/europe_land_iacs_prep). These will be regularly updated as we collect additional data throughout the Europe-LAND project period.

## Technical Validation

To validate the data, we verified that the number of records and the total area were preserved between the input data and the harmonised datasets. Furthermore, we compared the spatial coverage of the data and the number of farms with the official numbers on the total utilised agricultural area and number of farms per member state (Table 3). In most cases, the national statistics on agricultural area closely align with the area of the GSA data. Where smaller or larger disagreements occur, these can result from differences in the methods applied to produce the data. National statistics are often based on surveys, extrapolation of samples, or farm registers. GSA data, in contrast, is based on self-reporting by farmers or farmers’ consultants. A second reason for the discrepancy between the GSA data and national statistics can arise from the definition of the utilised agricultural area, which includes or excludes land-use categories that exist in some GSA data, e.g. landscape elements, forestry or farm roads, buildings or yards. Third, the number of farms can be inflated in cases where the data are recorded separately for subregions of a member state (e.g., federal states). In these cases, the farm identifiers may not be uniform across all the subregions and applicants that apply for subsidies in different regions can be recorded multiple times^22^. Finally, GSA data are only reported for farms that are eligible for EU CAP subsidies and that also apply for the subsidies. Some farms are smaller than the minimum areas required to be eligible for EU subsidies (the minimum eligible area varies across member states); similarly, a small share of farmers in some member states may be unwilling to apply for subsidies for various reasons.

**Table 3:** Comparison of the data content regarding spatial coverage and number of farms with official statistics.

Member state	Total area of data [1,000 ha]	Total utilised agricultural area in official statistics [1,000 ha]	Number of farms in data	Number of farms in official statistics
Austria	3,198	2,647	—	110,780
Belgium – Flanders	690	625	—	23,220
Bulgaria	3,884**	5,001**	—	132,740
Croatia	1,186	1,506	—	143,920
Czechia	5,792**	3,534**	35,206**	28,910
Denmark	2,658	2,620	37,185	37,090
Estonia	973	985	3,693	11,370
Finland	2,332	2,270	—	45,630
France	27,998	28,690	—	393,030
Germany –Brandenburg	1,336	1,437	—	5,410
Germany –Lower Saxony	2,572**	2,759*	—	—
Germany –North Rhine-Westphalia	1,492	1,603	—	—
Ireland	11,274	4,511	125,215	130,190
Latvia	1,782*	1,970*	—	68,980
Lithuania	2,913***	2,872**	—	132,080
Netherlands	1,878	1,814	—	52,640
Portugal	6,817	3,970	169,522	290,230
Slovakia	1,833	1,910	—	19,630
Slovenia	470	484	—	72,470
Spain	38,501*	24,693*	608,380*	914,870
Sweden	3,020	3,006	—	58,790

The year of comparison is 2020. In the few cases when we did not receive data from 2020, we used the closest available year as indicated by the asterisks: *2022, **2023, ***2024. Sources^23–25^.

## Usage Notes

In addition to the data published in the data inventory, we store additional information that cannot be shared publicly due to GDPR and data-sharing restrictions in an extended database. The data include harmonised GSA data for six additional EU Member States: Italy, Greece, Poland, Hungary, Romania, and Cyprus. In Italy, data are administered at the regional level, and we currently hold data for three regions: Emilia-Romagna, Marche, and Toscana. For the other five member states, we have complete national coverage. Beyond the datasets already included for Belgium (Flanders) and Germany (Brandenburg, Lower Saxony and North Rhine-Westphalia), we have also collected and harmonised GSA data for Wallonia in Belgium, and for three additional German federal states: Saarland, Saxony-Anhalt, and Thuringia. Additionally, we have gathered extra information on farm-level indicators and organic farming. Specifically, we hold farm identifiers for 17 additional member states, federal states, or regions (Austria, Cyprus, Greece, Latvia, Netherlands, Romania, Sweden, Slovenia, Slovakia, Wallonia in Belgium, Brandenburg, Lower Saxony, Saxony-Anhalt, and Thuringia in Germany, and Emilia-Romagna, Marche, and Toscana in Italy,) and organic farming information for seven more member states, federal states, or regions (Greece, Netherlands, Sweden, Slovenia, Slovakia, Wallonia in Belgium, and Brandenburg, Lower Saxony, Saarland, Saxony-Anhalt, and Thuringia in Germany). Lastly, for Luxembourg and Malta, we have not been able to obtain GSA data; only LPIS data were available at the time of writing. As these datasets contain only reference parcels without detailed land-use information at the parcel level, they were not included in the inventory.

Other users interested in accessing similar datasets can reach out directly to the relevant national or regional authorities responsible for IACS. To obtain these data, we submitted written data requests, often followed by further communication to clarify specific data needs or technical details. Once obtained, the datasets can be harmonised into the EuroCrops HCAT using our crop-mapping tables.

## Data Availability

The code for the pre-processing of the data and the harmonisation workflow are published on GitHub (https://github.com/clejae/europe_land_iacs_prep).
